# Dramatic response to local radiotherapy in a refractory metastatic mediastinal yolk sac tumor patient harboring a germline *BRCA2* frameshift mutation: a case report

**DOI:** 10.1080/15384047.2022.2072635

**Published:** 2022-05-16

**Authors:** Xi Cheng, Haiming Yu, Jinying Li, Xiaona Han, Erhong Meng, Houqing Zhou, Dongliang Wang, Beifang Niu, Xiaotao Zhang

**Affiliations:** a Department of Radiation Oncology, The Affiliated Qingdao Central Hospital of Qingdao University, Qingdao, China; b Department of Scientific Research Project, ChosenMed Technology (Beijing) Co., Ltd, Beijing, China; c Department of Medicine, ChosenMed Technology (Beijing) Co., Ltd, Beijing, China; d Computer Network Information Center, Chinese Academy of Sciences, Beijing, China; e School of Computer Science and Technology, University of the Chinese Academy of Sciences, Beijing, China

**Keywords:** Mediastinal yolk sac tumors, radiotherapy, DNA-damage repair, *BRCA2*, case report

## Abstract

Mediastinal yolk sac tumors (YSTs) are highly aggressive germ cell tumors with an extremely poor prognosis. Radiotherapy plays an important role in the treatment of mediastinal YSTs. To maximize benefit from radiotherapy in patients with mediastinal YSTs, exploring functionally relevant biomarkers is essential. Previous studies have demonstrated that mutations in DNA-damage repair (DDR) genes, including *BRCA1/2*, potentially enhance sensitivity to radiotherapy in solid tumors. However, DDR-gene mutations, as possible predictive biomarkers for radiotherapy in primary mediastinal YSTs, have not yet been reported. Herein, we report a 29-year-old male patient with a refractory metastatic primary YST involving a germline frameshift mutation in the *BRCA2* gene (NM_000059.3: exon11: c.4563_4564delAT: L1522fs). During treatment alternation, the patient was found to respond poorly to chemotherapy with or without an immune checkpoint inhibitor but well to radiotherapy. Finally, the patient achieved approximately 17 months of overall survival. To the best of our knowledge, this case report is the first to describe a remarkable response to local radiotherapy in a patient with a refractory metastatic mediastinal YST involving a DDR-gene mutation (germline *BRCA2* frameshift variation). This case report provides insightful clues for precision radiotherapy in clinical practice.

## Background

Yolk sac tumors (YSTs), namely, endodermal sinus tumors, are highly aggressive germ cell tumors (GCTs) occurring in the yolk sac. YSTs are classified as gonadal and extragonadal tumors, according to their locations. Extragonadal YSTs are relatively rare. They often occur in the middle axis of the body, such as the brain, mediastinum, and retroperitoneum.^1^ Mediastinal YSTs are more popular in infancy and post-puberty.^2^

Currently, the standard regimen for YST is surgery following chemotherapy with bleomycin, etoposide, and cisplatin (BEP).^3,4^ However, due to high malignancy and the infeasibility of complete resection at the time of diagnosis, the prognosis of primary mediastinal YSTs is extremely poor, with a five-year survival rate of 40%–50% and six-month survival following relapse.^5^

Previous studies have demonstrated that patients with primary mediastinal YSTs tend to respond well to chemotherapy but poorly to radiotherapy. Certain cases have been reported to be successfully treated with radiotherapy.^6^ However, predictive biomarkers for precise radiotherapy in primary mediastinal YSTs have not yet been explored.

DNA-damage repair (DDR) is essential for the survival of both malignant and normal cells.^7^ Preclinical data have revealed that radiosensitivity is associated with DDR.^8^ Recently, studies have demonstrated that DDR-gene mutations potentially predict an enhanced response to radiotherapy in patients with diverse solid tumors.^9^

Herein, we report a male patient with a refractory metastatic primary mediastinal YST. The disease progressed rapidly while the patient received chemotherapy with/without toripalimab (PD-1 antibody). However, the patient was considerably responsive to local radiotherapy. Meanwhile, a germline *BRCA2* mutation was detected in the patient using whole-exome sequencing (WES).

## Case presentation

**Figure 1.**
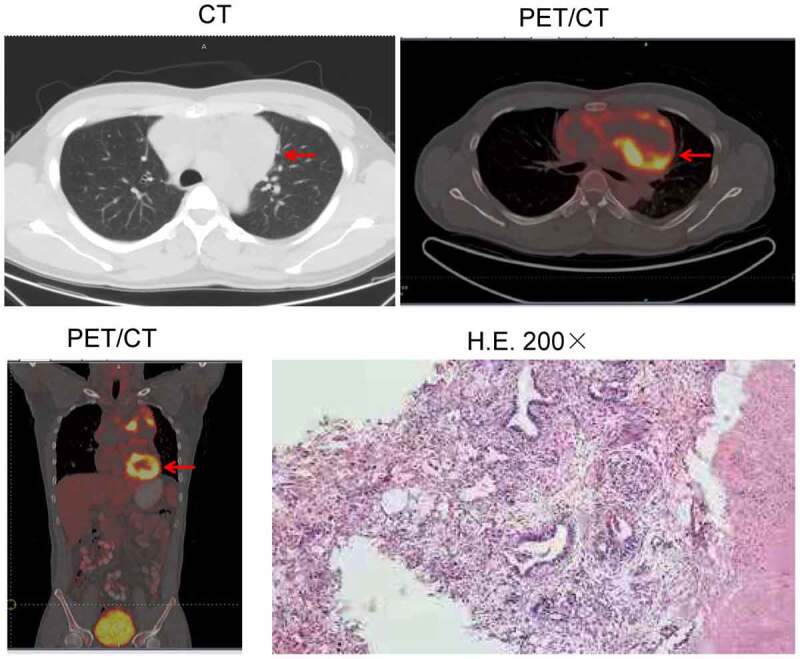
Radiographic imaging at diagnosis and pathological findings. Computed tomography (CT) and positron emission tomography/CT (PET/CT) revealed a space-occupying lesion in the anterior mediastinum, approximately 13.2 cm × 6.7 cm in size (red arrow). Surgical pathology revealed that the space-occupying lesion was a mediastinal yolk sac tumor (YST). CT: computed tomography; PET/CT: positron emission tomography/computed tomography; YST: yolk sac tumor; H.E.: hematoxylin and eosin

In January 2019, a 29-year-old male patient was admitted to our department due to paroxysmal cough, chest pain, tightness, and suffocation after activity. Thoracic computed tomography (CT) revealed an anterior mediastinal mass, approximately 13.2 cm × 6.7 cm in size. The level of alpha-fetoprotein (AFP) in the serum was 2,010.4 ng/mL. Positron emission tomography-CT (PET/CT) revealed a space-occupying mass in the anterior mediastinum invading the pericardium. On March 28, 2019, percutaneous CT-guided biopsy was performed, and the pathology confirmed that the space-occupying mass in the anterior mediastinum was an YST. Thus, the patient was diagnosed with mediastinal YST invading the pericardium. From May 7, 2019, to May 31, 2019, the patient received neoadjuvant radiotherapy (DT40Gy/20f), and the tumor subsequently subsided to 12.4 cm × 7.2 cm in size one week later. On June 27, 2019, the patient underwent resection of the mediastinal tumor and thymus. However, some lesions were unresectable (R2 resection). The final diagnosis was stage IV mediastinal YST, metastasizing to the bilateral lungs (Figure 1). First-line chemotherapy involving two cycles of etoposide and cisplatin was administered on August 1, 2019, and August 31, 2019. Subsequently, the anterior upper mediastinal mass enlarged, and multiple nodules in the lower lobe of the left lung were observed. Thus, the patient’s clinical response was evaluated as progressive disease (PD). On September 26, 2019, the treatment was alternated to second-line combined chemotherapy with carboplatin and paclitaxel. After completing two cycles of second-line therapy, the response was still considered PD. Therefore, the patient underwent third-line therapy combined with radiotherapy (DT50Gy/25f) on the lesion in the left lower lobe (one cycle of cisplatin and toripalimab [a PD-1 inhibitor, 240 mg, twice]). Thoracic CT performed in January 2020 revealed that all the lesions had progressed, except the lesion that had metastasized to the left lower lobe, which had been treated with local radiotherapy. On February 7, 2020, the patient was admitted to the Department of Respiratory due to cough, chest tightness, suffocation, and left-arm pain. On February 26, 2020, the level of AFP in the serum was 43,476.3 ng/mL. Moreover, thoracic CT revealed that the metastases in the lungs and pleura were increasingly enlarged. Therefore, the patient’s clinical response was regarded as PD.

Computed tomography (CT) and positron emission tomography/CT (PET/CT) revealed a space-occupying lesion in the anterior mediastinum, approximately 13.2 cm × 6.7 cm in size (red arrow). Surgical pathology revealed that the space-occupying lesion was a mediastinal yolk sac tumor (YST). CT: computed tomography; PET/CT: positron emission tomography/computed tomography; YST: yolk sac tumor; H.E.: hematoxylin and eosin.

**Figure 2.**
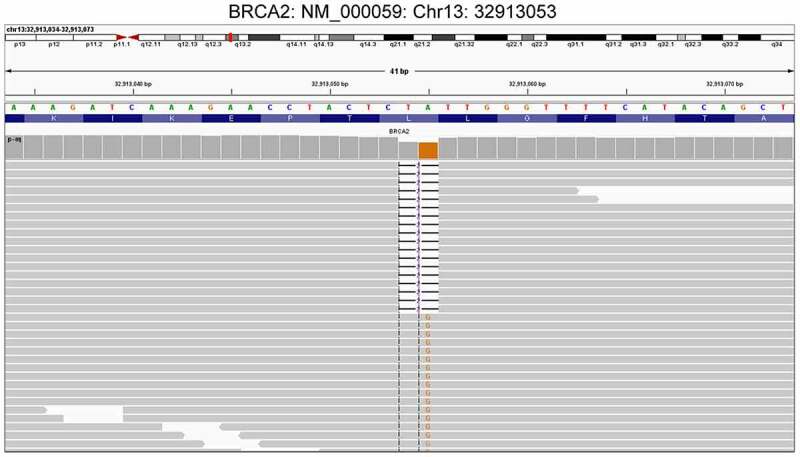
Sequencing reads of *BRCA2* shown by the IGV.A germline frameshift mutation in the BRCA2 gene (NM_000059.3: exon 11: c.4563_4564delAT: L1522fs) was detected in the patient by WES. The MAF was 50%. IGV: Integrative Genomics Viewer; WES: whole-exome sequencing; bp: base pairs; MAF: mutant allele frequency

To establish personalized treatment strategies, the tumor tissues obtained from the patient through abdominal biopsy and peripheral blood were subjected to WES on February 1, 2020. The results revealed that the patient harbored a germline *BRCA2* frameshift mutation (NM_000059.3: exon11: c.4563_4564delAT: L1522fs), with an allele frequency of 50%, and somatic *ERBB3* mutation (NM_001982.3: exon27: c.3213_3214delTT: S1072fs), with an allele frequency of 25%. The somatic and germline mutations of the patient are shown in Tables 1 and 2, respectively. The sequencing reads of *BRCA2* are shown in Figure 2. Furthermore, the patient was found to exhibit low microsatellite instability (MSI-L: 5.32%), medium tumor mutational burden (TMB-M: 2.64 Muts/Mb), low tumor neoantigen burden (TNB-L: 0.38 Neos/Mb), and a strongly positive loss-of-heterozygosity (LOH) status of the human leukocyte antigen (HLA) (Table 1).

**Table 1.** Somatic mutations of the patient (to be continued)

Gene	Transcript	Exon	Nucleotide change	Alteration	Mutant allele frequency
*AKIRIN1*	NM_024595.2	Exon 3	c.403C>T	R135*	12.50%
*AMELY*	NM_001143.1	Exon 2	c.52_53delCCinsTG	P18C	6.84%
*ANKRD36C*	NM_001310154.1	Exon 84	c.5957C>T	A1986V	4.65%
*ARFGEF3*	NM_020340.4	Exon 10	c.917G>C	G306A	10.93%
*ARID1B*	NM_001346813.1	Exon 20	c.6627G>T	M2209I	28.99%
*ARID1B*	NM_001346813.1	Exon 20	c.6628G>T	A2210S	29.04%
*BMP2K*	NM_198892.1	Exon 7	c.811G>T	V271F	14.02%
*BUD31*	NM_003910.3	Exon 5	c.224_225delAT	Y75fs	15.91%
*CBL*	NM_005188.3	Exon 8	c.1149A>G	I383M	58.57%
*CD300A*	NM_007261.3	Exon 2	c.119A>G	K40R	4.85%
*CD300A*	NM_007261.3	Exon 2	c.121G>T	E41*	4.95%
*CFAP74*	NM_001304360.1	Exon 19	c.2206C>T	P736S	20.51%
*COL4A3*	NM_000091.4	Exon 51	c.4872C>A	Y1624*	21.74%
*CXorf38*	NM_144970.2	Exon 1	c.193_194delCGinsAT	R65M	27.14%
*DAB2*	NM_001343.3	Exon 12	c.1552T>A	S518T	10.47%
*ELFN2*	NM_052906.4	Exon 3	c.293C>A	A98D	2.86%
*ERBB3*	NM_001982.3	Exon 27	c.3213_3214delTT	S1072fs	25.00%
*F8*	NM_000132.3	Exon 14	c.3949T>A	S1317T	45.00%
*FAM127B*	NM_001078172.1	Exon 1	c.37delG	A13fs	20.38%
*FASN*	NM_004104.4	Exon 7	c.841G>A	G281R	11.36%
*FBN3*	NM_001321431.1	Exon 9	c.901G>A	G301R	5.23%
*FCGBP*	NM_003890.2	Exon 7	c.3896C>T	A1299V	6.56%
*FGD6*	NM_018351.3	Exon 14	c.3469G>A	V1157I	12.90%
*FREM1*	NM_144966.5	Exon 27	c.4943G>A	G1648E	2.61%
*FRMD1*	NM_024919.4	Exon 6	c.723C>A	S241R	11.98%
*GABRR1*	NM_002042.4	Exon 4	c.340G>T	V114F	13.11%
*GRK4*	NM_182982.2	Exon 5	c.416C>G	S139C	24.50%
*HOXC11*	NM_014212.3	Exon 1	c.336G>C	E112D	6.31%
*HOXC11*	NM_014212.3	Exon 1	c.337A>T	I113F	5.50%
*IFIT1*	NM_001270927.1	Exon 3	c.827delA	K276fs	2.38%
*INPP4B*	NM_001331040.1	Exon 6	c.242_243delCCinsAA	T81K	19.44%
*JAK3*	NM_000215.3	Exon 16	c.2085_2089delTCTCC	C695fs	12.20%
*KCNA3*	NM_002232.4	Exon 1	c.1147G>A	G383R	12.41%
*KCNJ15*	NM_001276435.1	Exon 5	c.496G>A	A166T	2.19%
*KIF21A*	NM_001173464.1	Exon 21	c.2935_2936delGAinsTT	D979F	4.26%
*KNTC1*	NM_014708.4	Exon 3	c.191A>G	D64G	3.65%
*KRT2*	NM_000423.2	Exon 6	c.1201C>T	R401C	4.59%
*LAMA1*	NM_005559.3	Exon 51	c.7279A>C	N2427H	6.00%
*MUC6*	NM_005961.2	Exon 31	c.4889_4890delCAinsAG	T1630K	4.42%
*MYCBP2*	NM_015057.4	Exon 56	c.8877_8887delTGTGGATGAAG	S2959fs	17.39%
*MYO1G*	NM_033054.2	Exon 2	c.134dupT	L46fs	17.78%
*NEMP1*	NM_001130963.1	Exon 8	c.1055_1056delAGinsGT	E352G	16.36%
*NEO1*	NM_002499.3	Exon 18	c.2828T>C	M943T	12.73%
*NPAP1*	NM_018958.2	Exon 1	c.1734G>T	M578I	12.50%
*OBSCN*	NM_001271223.2	Exon 107	c.25366+1G>T	Splicing	13.51%
*OR4B1*	NM_001005470.1	Exon 1	c.703C>T	L235F	16.90%
*PBX1*	NM_002585.3	Exon 1	c.141A>C	L47F	17.05%
*PBX1*	NM_002585.3	Exon 1	c.142C>T	Q48*	16.09%
*PCYT1B*	NM_004845.4	Exon 4	c.396A>T	R132S	19.57%
*PEX5L*	NM_016559.2	Exon 7	c.647C>A	S216Y	11.81%
*PIGQ*	NM_148920.2	Exon 6	c.1116C>G	H372Q	21.74%
*PLEKHA5*	NM_001256470.1	Exon 12	c.1352G>T	S451I	5.93%
*PMF1-BGLAP*	NM_001199661.1	Exon 2	c.190T>C	C64R	3.21%
*PRPF31*	NM_015629.3	Exon 2	c.71G>A	G24E	10.81%
*PTPRO*	NM_030667.2	Exon 16	c.2560G>T	E854*	7.82%
*RNF144B*	NM_182757.3	Exon 2	c.5G>T	G2V	3.77%
*RRAS2*	NM_012250.5	Exon 1	c.68G>A	G23D	15.56%
*RRNAD1*	NM_015997.3	Exon 1	c.36G>C	E12D	8.50%
*RRNAD1*	NM_015997.3	Exon 1	c.37G>T	G13W	8.50%
*RSBN1*	NM_018364.4	Exon 3	c.1438G>C	V480L	12.20%
*SATB1*	NM_001195470.2	Exon 4	c.420delT	P141fs	8.10%
*SH2B3*	NM_005475.2	Exon 2	c.250_257delGCGCCGGG	A84fs	15.38%
*SLC4A3*	NM_001326559.1	Exon 4	c.469dupC	H157fs	9.09%
*SNAPC2*	NM_003083.3	Exon 5	c.881dupC	A295fs	2.44%
*SPNS3*	NM_182538.4	Exon 11	c.1385G>C	G462A	13.04%
*SRGAP1*	NM_020762.3	Exon 20	c.2407G>A	D803N	43.75%
*TIMM10B*	NM_012192.3	Exon 3	c.155G>T	C52F	15.27%
*TNKS1BP1*	NM_033396.2	Exon 6	c.2697C>A	S899R	21.15%
*TP53*	NM_000546.5	Exon 7	c.695T>C	I232T	49.30%
*TPST2*	NM_001008566.1	Exon 3	c.693G>C	E231D	2.20%
*UBA2*	NM_005499.2	Exon 10	c.938_939delTAinsAT	L313H	11.28%
*ZFPL1*	NM_006782.3	Exon 5	c.479A>T	N160I	14.29%
*ZFR*	NM_016107.4	Exon 20	c.3204A>T	K1068N	21.05%
*ZNF695*	NM_020394.4	Exon 4	c.542A>G	E181G	13.79%
*MSI-L*	5.32%				
*TMB-M*	2.64 Muts/Mb				
*TNB-L*	0.38 Neos/Mb				
*HLA LOH*	strongly positive

**Table 2.** Germline mutation of the patient

Gene	Transcript	Exon	Nucleotide change	Alteration	Mutant allele frequency
*BRCA2*	NM_000059.3	Exon 11	c.4563_4564delAT	L1522fs	50.0%

A germline frameshift mutation in the BRCA2 gene (NM_000059.3: exon 11: c.4563_4564delAT: L1522fs) was detected in the patient by WES. The MAF was 50%. IGV: Integrative Genomics Viewer; WES: whole-exome sequencing; bp: base pairs; MAF: mutant allele frequency.

**Figure 3.**
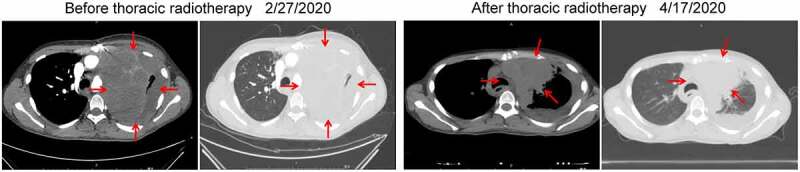
CT imaging before and after thoracic radiotherapy. After local radiotherapy, the lesions that had metastasized to the left lung significantly subsided compared with that before local radiotherapy. CT: computed tomography

Between March 4, 2020, and March 31, 2020, the patient underwent fourth-line therapy combined with left thoracic radiotherapy (95% of PGTVinner: 64.8Gy/3.6Gy/18f; 95% of PGTV: 43.2Gy/2.4Gy/18f) and oral olaparib (a poly [ADP-ribose] polymerase [PARP] inhibitor, 200 mg, bid). Thereafter, the patient’s asphyxia was significantly relieved, and thoracic CT revealed that the lesions that had metastasized to the left lung had subsided remarkably, indicating partial remission (PR, Figure 3). However, the patient complained of pain in the right hip. On March 25, 2020, magnetic resonance imaging (MRI) of the sacroiliac joints exhibited new multiple lesions in the pelvis. Between April 3, 2020, and April 25, 2020, the patient underwent radiation therapy of the pelvis (95% of PGTV: 37.5Gy/2.5Gy/15f). Subsequently, the pain in the right hip was alleviated, and the metastases in the pelvis subsided from 10 cm × 7 cm to 10 cm × 5.5 cm in size. Thus, the patient’s clinical response was evaluated as stable disease (SD).

After local radiotherapy, the lesions that had metastasized to the left lung significantly subsided compared with that before local radiotherapy. CT: computed tomography.

**Figure 4.**
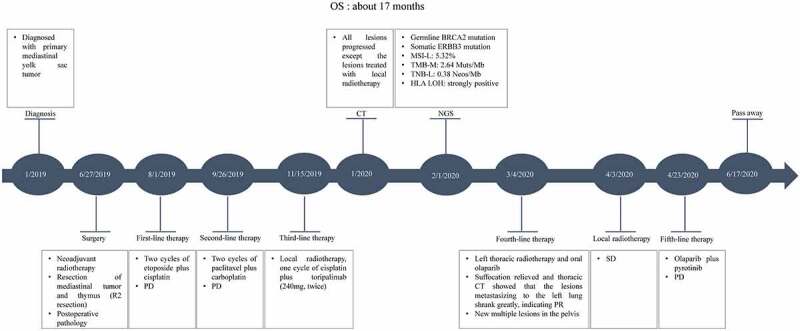
Case timeline. Timeline of diagnosis, NGS, and treatment in the patient. NGS: next-generation sequencing

On April 23, 2020, the patient underwent fifth-line therapy combined with oral olaparib (100 mg, bid) and pyrotinib (320 mg, qd). Seven days later, these two drugs were discontinued due to severe pneumonia. On May 17, 2020, the patient suddenly developed left-limb paralysis, and cerebral CT demonstrated that the tumor had metastasized to the right frontal parietal lobe with intratumoral hemorrhage. One month after symptomatic treatment, the patient died from respiratory and circulatory failure. The overall survival (OS) was approximately 17 months. The patient’s timeline is shown in Figure 4.

Timeline of diagnosis, NGS, and treatment in the patient. NGS: next-generation sequencing.

## Discussion

Herein, we report a patient with a refractory metastatic primary mediastinal YST. From the initial neoadjuvant radiotherapy to the subsequent multi-line therapy, the patient’s condition progressed rapidly upon receiving chemotherapy with/without toripalimab. However, the patient responded well to local radiotherapy, achieving PR. Moreover, a germline frameshift mutation in the *BRCA2* gene was detected in the patient through WES. To the best of our knowledge, this case report is the first to describe a dramatic response to radiotherapy in a refractory metastatic mediastinal YST patient harboring a germline *BRCA2* mutation.

In this study, we selected WES to detect the mutations in the patient, as it is a widely used next-generation sequencing (NGS) method that sequences all the protein-coding regions of the genome. The human exome represents < 2% of the genome; nevertheless, it contains up to 85% of known disease-associated variants,^10^ rendering WES a cost-effective alternative to whole-genome sequencing (WGS). In contrast to targeted gene sequencing, which analyzes one gene or small groups of related genes at a time, WES can analyze all the exons or coding regions of thousands of genes simultaneously. However, WES requires a longer time and more complex bioinformatics to analyze large amounts of sequencing data.

Radiotherapy plays a vital role in the management of mediastinal YSTs.^6^ To maximize benefit from radiotherapy, exploring functionally relevant biomarkers is essential. Previous studies have demonstrated that mutations in DDR genes, including *BRCA1/2*, potentially enhance radiosensitivity in patients with diverse solid tumors.^9^ However, DDR-gene mutations, as predictive biomarkers for radiotherapy in primary mediastinal YSTs, have not yet been explored.

Radiosensitivity depends on multiple factors, such as tumor histology, radiation dose, and intrinsic radiosensitivity of tumor cells, among others.^8,11–14^ Intrinsic radiosensitivity of tumor cells is the most important factor among them. As radiotherapy induces cell death predominantly via the generation of DNA double-strand breaks (DSBs), DDR can undoubtedly affect the radiosensitivity of cancer cells.^15^

The DDR system repairs diverse forms of DNA damage via eight pathways to properly protect the genome’s integrity. The eight pathways include mismatch repair (MMR), base-excision repair (BER), nucleotide-excision repair (NER), homologous-recombination repair (HRR), non-homologous end joining (NHEJ), check point factors (CPAs), Fanconi anemia (FA), and translesion DNA synthesis (TLS).^16–20^ The HRR and NHEJ pathways are responsible for DSBs, BER repairs single-strand breaks (SSBs),^21^ and the MMR pathway repairs DNA insertion/deletion corrections.^22^

*BRCA1* and *BRCA2* are two key molecules mediating the HRR pathway. The mutation of these two genes potentially disrupts DSB repair.^23^ As irradiation induces cell death predominantly through the generation of DNA DSBs, malignancies harboring a *BRCA1* or *BRCA2* mutation tend to respond well to ionizing radiation due to defects in the HRR pathway.^24^ Currently, substantial evidence supporting the role of *BRCA* in radiosensitivity has been obtained from preclinical data. For example, human cells with *BRCA1* and *BRCA2* variations contribute to enhanced radiosensitivity through an impaired proliferative capacity after irradiation.^25^ Moreover, transfecting wild-type *BRCA1* into *BRCA1^−/−^* human breast cancer cells potentially decreases the sensitivity of irradiation and increases the efficiency of DSB repair.^26^ Additionally, BRCA-deficient ovarian cancer cell lines are more sensitive to irradiation than parent cell lines.^27^ Recently, a study by Kim et al. demonstrated that solid tumors harboring *BRCA1/2* variations exhibited increased sensitivity to radiotherapy compared to those without these alterations.^9^ Our case is consistent with the findings of Kim et al. in this regard.

In addition to the germline *BRCA2* mutation, we also detected another mutation in the DDR gene: somatic *TP53* (NM_000546.5: exon7: c.695T>C: I232T). *TP53* is a core component of CPAs.^28^ However, DNA DSBs caused by irradiation are predominantly repaired via HRR and NHEJ repair pathways. To the best of our knowledge, related literature on the relationship between radiosensitivity and the CPA pathway is lacking.

Currently, the standard treatment for mediastinal YST is neoadjuvant chemotherapy combined with residual-tumor resection. Because the tumor is usually enlarged upon diagnosis and characterized by fibrous adhesion to adjacent organs, complete removal of the residual tumor after neoadjuvant chemotherapy is challenging, leading to a poor disease prognosis.^5^ Neoadjuvant chemotherapy represents the treatment backbone for mediastinal YSTs, and platinum-based chemotherapy is recommended for the initial treatment scheme.^29^ The common scheme comprises 4–6 cycles of cisplatin, etoposide, and ifosfamide (VIP) or BEP. Platinum-based chemotherapy enables up to 50% of patients to achieve long-term survival.^5,30^ Previous case reports have demonstrated postoperative pathological complete remission (pCR) after R0 resection following neoadjuvant chemotherapy.^5,31,32^ However, the current patient was not responsive to chemotherapy, and the reasons remain unclear.

In addition, the patient was refractory to ICI therapy. The possible underlying mechanisms might have been MSI-L, TNB-L, and strongly positive HLA LOH (Table 1), which indicates the simultaneous deletion of three genes: HLA-A, B, and C. HLA LOH is a common cause of immune escape. The presentation of new antigens by antigen-presenting cells through the antigens of HLA class I molecules plays a key role in cellular immunity. HLA LOH leads to a reduction in antigen presentation, thus promoting immune escape.^33^

Previous studies have revealed that *BRCA1*-mutation carriers are at an increased risk of breast, ovarian, prostate, and colon cancers, whereas those of the *BRCA2* mutation are at a higher risk of male breast, pancreatic, and prostate cancers.^34^ The pathogenesis of YSTs remains unclear. However, the germline *BRCA2* frameshift mutation in this patient should be closely related to the etiology. *BRCA2* germline mutations lead to defects in the HRR pathway, which is responsible for DSB repair. PARP inhibitors potentially inhibit SSB repair mediated by PARP-1 (i.e., the BER pathway), thus increasing the accumulation of DNA strand breaks and promoting genomic instability and apoptosis. Previous studies have demonstrated that PARP inhibitors can effectively destroy tumors with defective *BRCA* genes as well as those in testicular cancer cell lines with low homologous recombination proficiency according to the concept of synthetic lethality. To date, the FDA approved Olaparib for the treatment of ovarian, breast, and prostate cancers involving germline *BRCA* mutations. In addition, Olaparib demonstrated remarkable clinical efficacy in the treatment of germline *BRCA*-mutated pancreatic cancer and small-cell lung cancer (SCLC).^35^

Furthermore, a study by Yue Bi et al. demonstrated that Olaparib is potentially useful as an effective radiosensitizer in *BRCA1*-deficient high-grade serous ovarian carcinomas using a preclinical model.^36^ This led us to hypothesize that PARP inhibitors may be used as potential radiosensitizers to enhance the sensitivity of radiotherapy in *BRCA2*-deficiency YST. As expected, this case exhibited a favorable response to the combination of local radiotherapy with Olaparib.

Notwithstanding, this study has a limitation. We did not perform an extra set of experiments with real-world samples to strengthen the conclusion, i.e. we did not develop cellular models to clarify how the specific *BRCA2* mutation impacts BRCA2 function, especially in relation to X-ray sensitivity.

## Conclusion

To the best of our knowledge, our case report is the first to describe a dramatic response to radiotherapy in a refractory metastatic mediastinal YST patient harboring a germline *BRCA2* mutation. This study potentially provides insightful clues for precision radiotherapy in clinical practice. Further studies that clarify how the specific *BRCA2* mutation impacts BRCA2 function are warranted in order to strengthen the conclusion.

## Data Availability

All the data supporting the findings are available upon reasonable request from the corresponding author (Xiaotao Zhang).

## Abbreviations

YSTs: yolk sac tumors; GCTs: germ cell tumors; BEP: cisplatin, etoposide, and bleomycin; DDR: DNA-damage repair; WES: whole-exome sequencing; CT: computed tomography; AFP: alpha-fetoprotein; PET: positron emission tomography; PD: progressive disease; MSI-L: low microsatellite instability; TMB-M: medium tumor mutational burden; TNB-L: low tumor neoantigen burden; LOH: loss of heterozygosity; HLA: human leukocyte antigen; PARP: poly (ADP-ribose) polymerase; PR: partial remission; MRI: magnetic resonance imaging; SD: stable disease; OS: overall survival; NGS: next-generation sequencing; WGS: whole-genome sequencing; DSBs: DNA double-strand breaks; MMR: mismatch repair; BER: base-excision repair; NER: nucleotide-excision repair; HRR: homologous-recombination repair; NHEL: non-homologous end joining; CPAs: check point factors; FA: Fanconi anemia; TLS: translesion DNA synthesis; SSBs: single-strand breaks; VIP: cisplatin, etoposide, and ifosfamide; pCR: pathological complete remission; SCLC: small-cell lung cancer.
